# Phenotypic heterogeneity in females with X-linked Alport syndrome 

**DOI:** 10.5414/CN108561

**Published:** 2015-08-07

**Authors:** Samuel C. Allred, Karen E. Weck, Adil Gasim, Amy K. Mottl

**Affiliations:** 1 University of North Carolina School of Medicine, Chapel Hill, NC,; 2 Department of Pathology and Laboratory Medicine,; 3 Department of Genetics, and; 4 University of North Carolina Kidney Center, UNC School of Medicine, Chapel Hill, NC,USA

**Keywords:** Alport syndrome, X-linked, X-chromosome inactivation, females, phenotype

## Abstract

Aims: X-linked Alport syndrome (AS) is a monogenic inherited disorder of type IV collagen, a structural protein in the kidney and cochlea. Males typically exhibit a severe phenotype with end-stage renal disease (ESRD) and/or deafness by early adulthood. Because of the presence of two X chromosomes, females often have a less severe phenotype and hence the diagnosis of AS is often not considered. Herein, we present a case of an adolescent girl with proteinuria and hematuria in the setting of a strong family history of AL. Case report: The mother and maternal aunt of the proband had both presented with dipstick positive hematuria and proteinuria at age 8 years. These girls were not evaluated by nephrology until mid-adolescence when they had worsening creatinine levels. Kidney biopsy in the younger sister demonstrated segmental glomerulosclerosis with segmental thinning and lamination of the glomerular basement membrane, consistent with AS. Kidney biopsy in the older sister was performed just prior to the need for renal replacement therapy and showed only global glomerulosclerosis. Both sisters were transplanted by the age of 20 years. Their mother subsequently developed ESRD at age 53 years. With the advent of genetic testing, the proband and her family were brought in for evaluation. It had been assumed this family of AS had autosomal dominant transmission, however, genetic testing of the proband was positive for a splice site mutation of *COL4A5* located on the X-chromosome. Sequencing of genes *COL4A3*, *COL4A4*, and *COL4A6* were negative for mutation. Conclusions: The current case report demonstrates the importance of considering skewed X-inactivation in females who exhibit signs or symptoms of X-linked disorders.

## Introduction 

Alport Syndrome (AS) is a Mendelian genetic disorder characterized by hematuria, progressive renal dysfunction and hearing loss. The prevalence of AS has been estimated at 1 : 5,000 from a cohort in Utah [1], but was much less common (1 : 53,000) in a Northern European cohort [2]. X-linked Alport Syndrome (XLAS) was previously considered the mode of inheritance for the vast majority of AS [3], however, next generation sequencing has recently revealed that XLAS accounts for between 37 and 65% of AS, while autosomal dominant transmission accounts for ~ 26 – 31% [4, 5]. AS is a disease caused by mutation in one or more of the α-chains forming type IV collagen which is important to the structural integrity of basement membranes in the glomerulus, cochlea, and eye [6]. 

Type IV collagen is comprised of a set of six distinct α-chains, α1(IV) to α6(IV), which self-assemble to form three distinct heterotrimeric helices: α1α1α2, α3α4α5, and α5α5α6. XLAS is caused by defects in α5(IV) chain, resulting in either absent or aberrant a3α4α5 and α5α5α6 helices. In hemizygous males, progressive renal dysfunction and end-stage renal disease (ESRD) occurs in 90% of individuals by age 40 [7]. Hearing loss also occurs in 90% of males and is characterized by symmetrical deficits in detection of high frequency sounds, usually manifest by late childhood [3]. Ocular abnormalities are common in men, in whom ~ 15% exhibit anterior lenticonus which is associated with reduced visual acuity and cataracts [8]. Female carriers of XLAS typically have a less severe phenotype than males [7]. It is commonly known that the vast majority will still have microscopic hematuria and proteinuria, but the risk for worsening renal function and hearing loss is vastly underappreciated. The following case report underscores the need for recognition of a possible X-linked carrier state in females with prompt subspecialty referral to evaluate and delay the progression of disease. 

## Case report 

A 13-year-old girl was brought in for evaluation because of the finding of hematuria and proteinuria. The child’s creatinine was 0.7 mg/dL, and urine protein : creatinine ratio was 0.8 g/g. Both her mother and maternal aunt had been clinically diagnosed with AS during young adulthood and hence were presumed to be autosomal dominant. 

**Figure 1.**
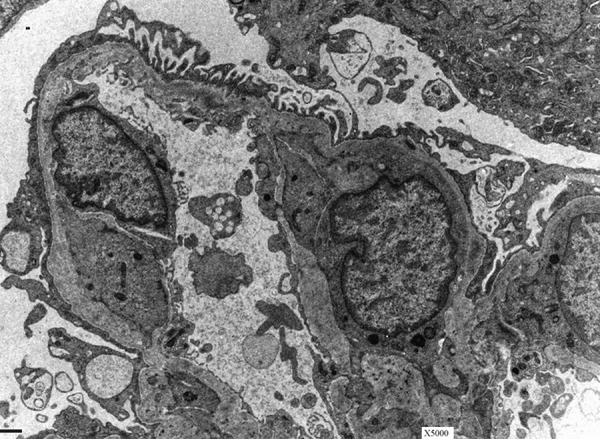
Electron micrograph from kidney biopsy of the proband’s mother demonstrating characteristic findings of Alport’s carrier state. The glomerular basement membranes have variable segmental lamination, creating a “basket weave” appearance and there is segmental foot process effacement with microvillus transformation.

The proband’s mother was first evaluated by nephrology at the age of 16 years. She was referred for evaluation of hematuria and proteinuria on urinary dipstick dating back to the age of 8 years old. Her blood pressure was 120/88 mmHg, and she had trace bipedal edema. Her creatinine was 1.4 mg/dL and 24-hour urine protein, 10.5 g. She underwent kidney biopsy for which light microscopy revealed 2/17 globally sclerosed glomeruli, 3/17 with segmental sclerosis and variable moderate to moderately severe interstitial fibrosis and tubular atrophy. Immunofluorescence microscopy was trace-positive for IgM only. Electron microscopy demonstrated segmental thinning and variable wrinkling of the glomerular basement membrane with variable segmental lamination. There were no electron-dense deposits or inclusions. There was variable effacement of epithelial foot processes with focal microvillous transformation (Figure 1). Immunofluorescent staining for collagen IV was not available at the time; however, all findings were consistent with AS. She was begun on an angiotensin converting enzyme inhibitor, but suffered a rapid deterioration in her renal function and was on hemodialysis after 12 months. 

The maternal aunt had also had a history of hematuria and proteinuria since the age of 8 years. At age 10, she was diagnosed with type 1 diabetes after presenting with diabetic ketoacidosis. It is unknown whether she was ever on an ACEI, and she had a rapid progression of renal failure with her creatinine rising from 0.9 mg/dL at age 16 years to 4.4 mg/dL 2 years later. Her 24-hour urine protein was 14 g. She did not have retinopathy. She underwent kidney biopsy at 18 years, with the finding of advanced global glomerulosclerosis; hence an exact etiology could not be ascertained. Given that the biopsy and renal disease progression were atypical for diabetes, her hematuria and proteinuria predated her diabetes, and her sister had proven AS, the primary etiology of her kidney failure was suspected to be autosomal dominant AS. She was started on dialysis less than 2 months later and was transplanted at the age of 21 years. 

The proband was hence brought in for genetic testing in an effort to avoid kidney biopsy. Although it had been assumed that the proband’s mother and maternal aunt had autosomal dominant AS, *COL4A5* was prioritized for testing given the absence of known disease in the maternal grandparents. *COL4A5* genetic analysis was performed using DNA mutation scanning by high resolution melting analysis (HRMA) of the 51 coding exons and flanking intronic regions of the *COL4A5* gene followed by sequencing of genomic regions positive by HRMA. A heterozygous splicing mutation in intron 8 was identified: *COL4A5* IVS8-1G>A. This novel mutation disrupts a conserved splice acceptor site and is therefore predicted to be deleterious. Although this mutation has not been previously described, splice site mutations typically result in defects in RNA splicing and result in an aberrant protein. Splicing mutations resulting in exon skipping have previously been described in association with AS [9]. Therefore these results are consistent with a diagnosis of X-linked AS. 

**Figure 2.**
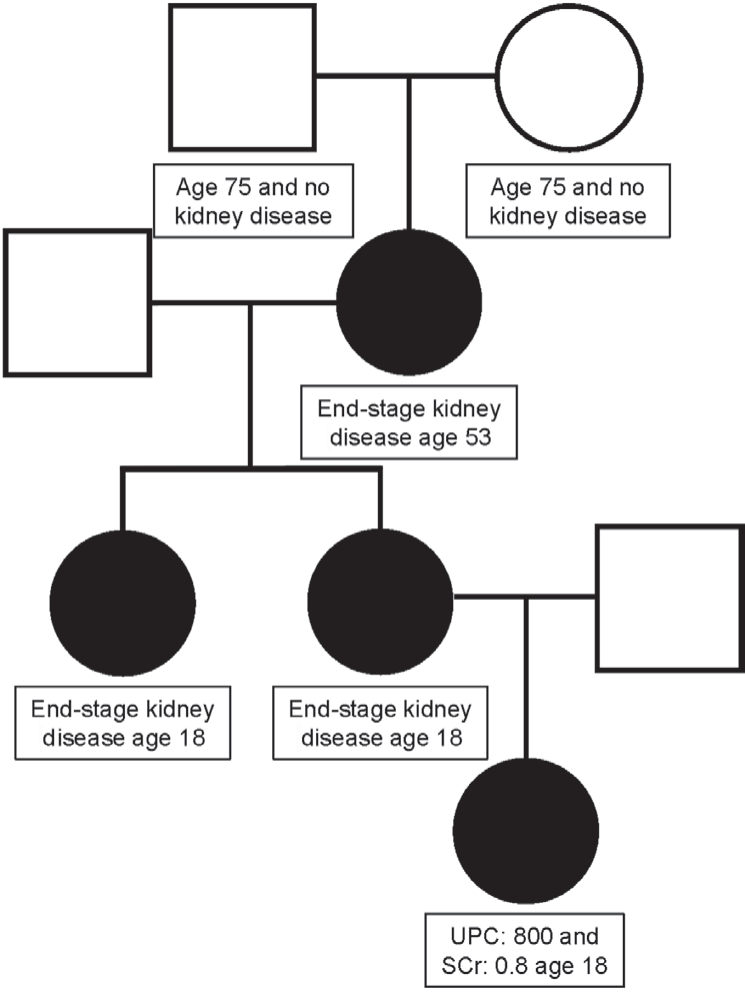
Pedigree for this case report of Alport syndrome; UPC = urine:protein creatinine ratio; SCr = serum creatinine.

Subsequent to genetic testing, the maternal grandmother was admitted to the hospital for pneumonia at age 53 years and was found to be in florid kidney failure. She had not sought prior medical care and did not know that she had kidney disease. At this point, the mother, maternal aunt, and maternal grandmother presented for next generation genetic testing of all three AS causing genes*: COL4A3, COLA4 COL4A5* and *COL4A6.* All three women had the same *COL4A5* IVS8-1G>A mutation as the proband. No other mutations were found. The parents of the maternal grandmother have both lived into their 70s and have had no known kidney disease. It is assumed that this was a spontaneous mutation in the maternal grandmother. The pedigree for this family is depicted in Figure 2. None of the women in the current report have opted to undergo hearing or ocular evaluation but have reported no clinically relevant hearing or visual defects. 

## Discussion 

Female “carriers” are frequently considered to be “protected” from X-linked disorders since they harbor a wild-type copy on the other X chromosome. Despite the wealth of our molecular understanding of X-inactivation and clinical documentation, X-linked disorders are often not considered in the differential diagnosis for females presenting with signs or symptoms of X-linked disease. Female carriers of XLAS exhibit extensive phenotypic heterogeneity with respect to age of onset and rapidity of disease progression. Studies have demonstrated that 12% of females develop ESRD by age 40, and 30 – 40% of female carriers will develop ESRD by age 60 [10]. Hearing loss also manifests in roughly 10% of XLAS female carriers by middle-age [10]. 

There is a significant degree of phenotypic heterogeneity amongst both males and females with XLAS. Heterogeneity in the hemizygous male is largely due to genotype-phenotype correlation, wherein more severe mutations in the protein result in more severe clinical manifestations [7]. Generally, nonsense mutations or large deletions typically result in earlier onset of disease with more rapid deterioration in renal function and hearing loss, versus missense or splice site mutations which often result in milder phenotypes [7]. In contrast, phenotypic heterogeneity in heterozygous females is less well explained by genotype-phenotype correlation [9]. Recently, evidence in a murine model has implicated skewing of X-inactivation as the likely mechanism underlying the variability of disease severity in female XLAS carries [11]. 

The distribution of sex chromosomes results in an inequality of gene copy number between males (XY) and females (XX). To circumvent this imbalance, females randomly inactivate one of the two X chromosomes in any cell line [12]. Since this is usually a stochastic event, females have a mosaic population of cells that express either the maternally or the paternally derived X chromosome, typically 50 : 50 [12]. Given the random nature of X-inactivation, a single organ may have a pattern that deviates or skews from the expected ratio, potentially leading to an imbalance as great as 80 : 20 [13]. Thus, unfavorable skewing of X-inactivation, such that the wild-type allele becomes silenced, may underlie the phenotypic variability seen in female carriers of X-linked disorders. Non-random skewing of X-inactivation has also been documented in which the diseased allele either confers a selective advantage or regulates the inactivation process [14]. 

Close monitoring and timely therapy in patients with X-linked AS, as well as carriers, can delay the progression of disease [15, 16]. Angiotensin-converting enzyme (ACE) inhibition has been shown to delay renal failure and prolong life expectancy in males with XLAS and in individuals with autosomal recessive AS [14]. AS carriers treated with renin-angiotensin-aldosterone system (RAAS) blockade have been noted to have a significantly later onset of ESRD than in those without RAAS blockade treatment [15]. Carriers of AS are often excluded from clinical trials, however, and clinical practice guidelines often focus on treating male patients [17]. 

This case report reflects the importance of recognizing the potential for clinical manifestations of XLAS in females throughout the lifespan. There is significant opportunity to impact the disease course of AS in the carrier state with aggressive blood pressure control and RAAS blockade and hence physicians should be cognizant of this when caring for patients. Natural history studies of clinical outcomes and the pattern of X-inactivation may help to predict disease progression in female carrier patients. 

## Conflict of interest 

The authors report no conflicts of interest. The authors alone are responsible for the content and writing of the paper.
